# Prevalence of *Helicobacter pylori* infection and antibiotic resistance profile in Armenia

**DOI:** 10.1186/s13099-019-0310-0

**Published:** 2019-06-08

**Authors:** Manik Gemilyan, Gagik Hakobyan, Lucie Benejat, Bujana Allushi, Davit Melik-Nubaryan, Harutyun Mangoyan, Amandine Laur, Emilie Daguerre, Eduard Grigoryan, Francis Megraud

**Affiliations:** 1 Yerevan State Medical University, 2 Koryunst, 0025 Yerevan, Armenia; 2 French National Reference Centre for Helicobacters, Bacteriology laboratory, Pellegrin Hospital, Bordeaux, France; 3 Vardanants Center for Innovative Medicine, Vardanants 18/1, 0010 Yerevan, Armenia; 4 Armenian Association of Gastroenterology, 5B Mashtotsave, 0015 Yerevan, Armenia; 5 INSERM 1053, University of Bordeaux, Bordeaux, France

**Keywords:** *Helicobacter pylori*, Seroprevalence, Gastric biopsies, Real-time PCR, Sequencing, Clarithromycin, Levofloxacin

## Abstract

**Background:**

The prevalence of *Helicobacter pylori* infection was never assessed in Armenia, nor was the prevalence of *H. pylori* resistance against the main antibiotics concerned, despite the fact that these data are fundamental to establish evidence-based recommendations for management of this infection. We aimed to fill this gap by assessing prevalence of *H. pylori* among adult population in Armenia and resistance of *H. pylori* strains to clarithromycin and levofloxacin.

**Methods:**

*Helicobacter pylori* seroprevalence was determined in 217 asymptomatic adult subjects submitted to a health checkup using an ELISA. Molecular methods were used to detect *H. pylori* in gastric biopsies from 91 adult dyspeptic patients [55 (60.4%) were positive] as well as the mutations associated with clarithromycin resistance by real-time PCR and with levofloxacin by sequencing the *gyr*A QRDR.

**Results:**

*Helicobacter pylori* seropositivity was found to be 41.5% globally and increased with age from 13.6% (age 18–25 years) to 83.3% (age > 65 years). Only two cases were found with a A2142/43G mutation causing clarithromycin resistance, and 6 cases showed mutations associated with levofloxacin resistance.

**Conclusions:**

*Helicobacter pylori* infection is estimated to be about 42% among adults in Armenia and the low clarithromycin resistance allows the use of the standard triple therapy as a first line therapy.

**Electronic supplementary material:**

The online version of this article (10.1186/s13099-019-0310-0) contains supplementary material, which is available to authorized users.

## Background

*Helicobacter pylori* (*H. pylori*) is a Gram-negative bacterium colonizing the stomach. Infection by *H. pylori* causes gastritis in all infected subjects [1, 2], as well as peptic ulcer disease and gastric cancer in a proportion of infected individuals [3].

The prevalence of *H. pylori* infection varies in different parts of the world, essentially due to the level of development which can partly be explained by promiscuity, low education and lack of hygiene facilities especially water [4]. According to a review that summarized studies involving populations with a large age span, among European countries, the percentage of population infected by *H. pylori* was lowest in Denmark (17%) and Switzerland (19%) and highest (> 80%) in Russia, Portugal and Estonia [5]. The prevalence of *H. pylori* infection in Africa, Asia and Latin America is generally higher than in Europe, and a trend towards decreasing prevalence was noted in several countries between 2009 and 2016 compared to 2000 and 2009 period [6]. However, Armenia has remained a “blank” spot on the *H. pylori* prevalence map, since no data have been published for the country.

Current guidelines on *H. pylori* management suggest to abandon the so-called standard triple therapy including a proton pump inhibitor (PPI) and two antibiotics: clarithromycin and amoxicillin or clarithromycin and metronidazole because of the high rate of clarithromycin resistance (> 15%) in most European regions except Northern Europe, or to perform antimicrobial susceptibility testing before starting treatment [2]. Levofloxacin-containing regimens can be used as a rescue therapy; however, resistance to fluoroquinolones is also frequent and is a major limiting factor for using such schemes. Given that no data were available on *H. pylori* resistance in Armenia, it was crucial to perform a survey to be able to make recommendations for first and second line treatments against this infection.

With current report we aimed to fill this gap by assessing prevalence of *H. pylori* among general adult population in Armenia and resistance of *H. pylori* strains to the main antibiotics known to be produce resistance, i.e. clarithromycin and levofloxacin.

## Methods

The recruitment of study participants was done during the period of December 12, 2017 to January 11, 2018, in two medical centers: “Vardanants Center for Innovative Medicine” (VMC) and “Armenia Republican Medical Center”. Written, informed consent was obtained from each patient included in the study.

### Determination of *H. pylori* prevalence

*Helicobacter pylori* prevalence was determined by serology. Adult subjects (age 18 years and older) presenting in VMC for routine health checkup according to their insurance plan were approached by medical volunteers who explained the study protocol and invite them to participate. In all subjects who signed the informed consent, 4 ml blood was drawn in addition to the amount intended for checkup. The blood was immediately centrifuged, and the serum decanted and maintained at + 4 °C. At the end of each day the sera were transferred in an ice box and placed in a freezer at − 80 °C. After collection of all sera, they were transferred frozen by courier service to Bordeaux, France were they were processed.

A commercially available ELISA (Enzygnost Anti *H. pylori* II IgG, Siemens, Munich, Germany) was used. This kit was previously evaluated by the French regulatory authorities (AFSSAPS) with an accuracy of 93.5% [95% CI 88.5–98.5], in comparison to invasive methods [7]. It was used according to the supplier’s recommendations.

### Determination of *H. pylori* resistance to antibiotics

Presence of *H. pylori* and its resistance to clarithromycin and levofloxacin were determined by molecular methods. At the Armenia Medical Center, all patients who presented for endoscopy with dyspeptic symptoms were invited to participate in the study. For those who signed the informed consent, an additional tissue sample was taken from the gastric antrum and was immediately placed into an empty Eppendorf tube and stored in a refrigerator (+ 4 °C). At the end of each day all tubes were transferred in an ice box and placed in a freezer at − 80 °C. After collection of all gastric biopsies, they were transferred frozen by courier service to Bordeaux, France where they were processed.

To detect *H. pylori* and its resistance to clarithromycin, an in-house real-time PCR was used. Briefly after DNA extraction the PCR was run in a LightCycler apparatus with specific primers and labelled probes [8]. For levofloxacin resistance, the Quinolone resistance determining region (QRDR) of the *gyr*A gene was amplified and sequenced by using the primers:

F-QRDR-Hpylo(GCGTATTTTGTATGCGATGC) and

R-QRDR-Hpylo(ACAAAATCAATGGTGTCTTTATCA).

Cycling conditions consisted of an initial denaturation at 95 °C for 3 min, 40 cycles of denaturation at 95 °C for 30 s, annealing at 58 °C for 30 s and elongation at 72 °C for 30 s, followed by a final extension at 72 °C for 5 min. PCR amplicons were examined by applying 5 µl on a 2% agarose gel and then purified using the ExoSAP-IT (Applied Biosystems, Foster City, CA, USA). Purified PCR products were sequenced with the BigDye Terminator Cycle Sequencing Reaction Kit v3.1 (Applied Biosystems) using the PCR primers as sequencing primers. Sequencing was accomplished with an ABI 3130 Genetic Analyzer (Applied Biosystems), and the obtained results were analysed with DNAbaser software (HeracleBiosoft, Germany).

### Statistics

Results of analyses were de-identified, entered into an Excel spreadsheet and analyzed using SPSS 20.0 software.

## Results and discussion

### Prevalence of *H. pylori* infection

**Fig. 1:**
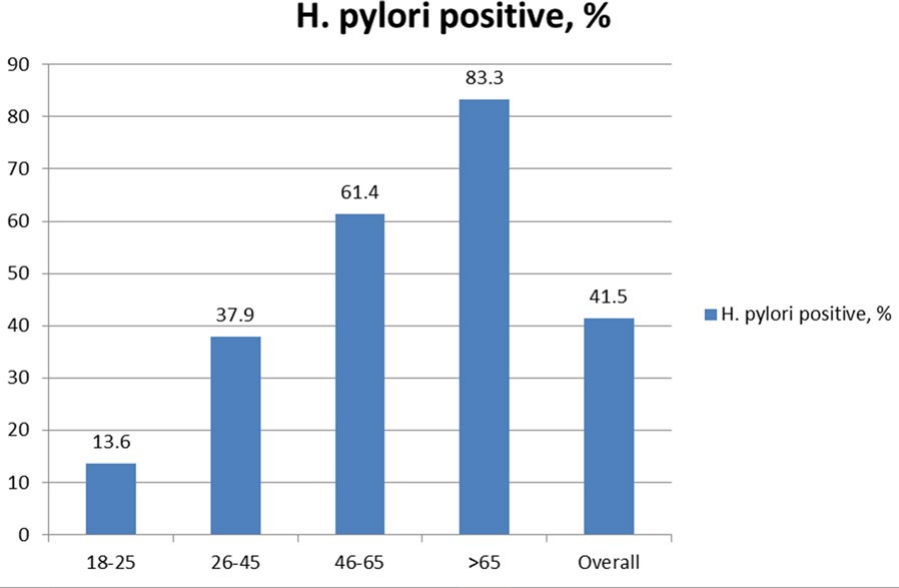
Distribution of *H. pylori* seropositivity (in percentages) among healthy individuals in Armenia according to age

The total number of study participants was 230, with a mean age 39.2 years ± 13.0 (range 18–74); 178 (77.4%) were female. Ninety subjects (39.1%) harbored *H. pylori* antibodies while 127 (55.2%) tested negative and 13 (5.7%) were in the grey zone. After eliminating doubtful results, among a total of 217 subjects the rate of positive antibody tests was 41.5%. The distribution of *H. pylori* seropositivity by age is presented in Fig. 1. The frequency of *H. pylori* seropositivity was 39.6% in males and 42.0% in females. The lowest rate (13.6%) was reported in the youngest age group (18–25 years), and the highest rate (83.3%) in the age group over 65 years. The prevalence steadily increased with age (Additional file 1).

It is interesting to see that the prevalence of *H. pylori* infection is less than 40% for those aged 26–45 year, and much lower than 90% for the higher age groups. However, this study has some limits, because a recruitment bias is possible since the individuals were recruited in only one center in the capital city Yerevan, and they were part of an insurance check-up which may indicate a higher socioeconomic level. The age distribution between the groups was also not balanced. Furthermore, despite the fact that the ELISA used was one of the best, it was not validated in Armenia and so it is possible, although unlikely, that the Armenian strains have a different antigenic profile.

### Prevalence of *H. pylori* resistance

The number of study participants providing antral biopsy specimens was 91, 64.8% were female. Analysis of gastric antral tissue specimens revealed that 55 (60.4%) of the patients with dyspeptic symptoms were infected by *H. pylori*. Of these, the mutations associated with clarithromycin resistance (A2142-43G) were found in 2 patients (3.6%), and with fluoroquinolone resistance (mutation N87K or D91Y) in 6 (12.8%), 3 of each (Additional file 2).

## Conclusions

These data suggest that Armenia ranks among countries with a low *H. pylori* macrolide resistance rate as well as a medium fluoroquinolone resistance rate. This gives an excellent background to suggest standard triple therapy with PPI, amoxicillin and clarithromycin as a first-line treatment option for *H. pylori* infection in Armenia [2]. However, triple therapy containing levofloxacin should not be offered as a first-line treatment because it is known that resistance to this antibiotic group can develop rapidly and levofloxacin-containing regimens are generally discouraged from being used as a first-line option. Despite the fact that culture and antibiogram could not be carried out, the detection of mutations by molecular methods provides a reliable substitute in which we can be confident [9]. Furthermore resistance against other antibiotics, e.g. amoxicillin, tetracycline and rifabutin, is seldomly found and does not necessitate a routine checkup [10]. Resistance to metronidazole has not been studied in Armenian population. However, if detected in vitro, it can be overcome by a prolonged treatment and adding bismuth to the eradication scheme.

## Additional files

**Additional file 1.** Contains all data on serological study results for *H. pylori* antibodies in serum of 230 subjects.

**Additional file 2.** Contains all data on macrolide and fluoroquinolone resistance mutation testing in gastric biopsy specimens of 91 patients.

## Abbreviations

AFSSAPS: French Health Products Safety Agency
ELISA: enzyme-linked immunosorbent assay
*H. pylori*: *Helicobacter pylori*
PCR: polymerase chain reaction
PPI: proton pump inhibitor
QRDR: quinolone resistance determining region
VMC: Vardanants Center for Innovative Medicine

## Availability of data and materials

All data generated or analysed during this study are included in this published article and its Additional files.
